# Combining Culture-Dependent and Independent Approaches for the Optimization of Epoxiconazole and Fludioxonil-Degrading Bacterial Consortia

**DOI:** 10.3390/microorganisms9102109

**Published:** 2021-10-07

**Authors:** Diogo A. M. Alexandrino, Ana P. Mucha, Maria Paola Tomasino, C. Marisa R. Almeida, Maria F. Carvalho

**Affiliations:** 1 CIIMAR—Interdisciplinary Centre of Marine and Environmental Research, University of Porto, Terminal de Cruzeiros do Porto de Leixões, Avenida General Norton de Matos s/n, 4450-208 Matosinhos, Portugal; amucha@ciimar.up.pt (A.P.M.); mtomasino@ciimar.up.pt (M.P.T.); calmeida@ciimar.up.pt (C.M.R.A.); mcarvalho@ciimar.up.pt (M.F.C.); 2 School of Medicine and Biomedical Sciences (ICBAS), University of Porto, Rua de Jorge Viterbo Ferreira 228, 4050-313 Porto, Portugal; 3 Faculty of Sciences, University of Porto (FCUP), Rua do Campo Alegre 790, 4150-171 Porto, Portugal

**Keywords:** culture-dependent approaches, defluorination, epoxiconazole, fludioxonil, persistent pesticides, metabarcoding, biodegradation

## Abstract

Epoxiconazole (EPO) and fludioxonil (FLU) are two widely used fluorinated pesticides known to be highly persistent and with high ecotoxicological potential, turning them into pollutants of concern. This work aimed to optimize two degrading bacterial consortia, previously obtained from an agricultural soil through enrichment with EPO and FLU, by characterizing the contribution of their corresponding bacterial isolates to the biodegradation of these pesticides using both culture-dependent and independent methodologies. Results showed that a co-culture of the strains *Hydrogenophaga eletricum* 5AE and *Methylobacillus* sp. 8AE was the most efficient in biodegrading EPO, being able to defluorinate ca. 80% of this pesticide in 28 days. This catabolic performance is likely the result of a commensalistic cooperation, in which *H. eletricum* may be the defluorinating strain and *Methylobacillus* sp. may assume an accessory, yet pivotal, catabolic role. Furthermore, 16S rRNA metabarcoding analysis revealed that these strains represent a minority in their original consortium, showing that the biodegradation of EPO can be driven by less abundant phylotypes in the community. On the other hand, none of the tested combinations of bacterial strains showed potential to biodegrade FLU, indicating that the key degrading strains were not successfully isolated from the original enrichment culture. Overall, this work shows, for the first time, the direct involvement of two bacterial species, namely *H. eletricum* and *Methylobacillus* sp., in the biodegradation of EPO, while also offering insight on how they might cooperate to accomplish this process. Moreover, the importance of adequate culture-dependent approaches in the engineering of microbial consortia for bioremediation purposes is also emphasized.

## 1. Introduction

Pesticides represent challenging compounds for microbial degradation due to their chemical heterogeneity and bioactive profiles [1]. Fluorinated pesticides further extend this challenge by combining different persistent functional groups with the high recalcitrance of fluorine substitution in their molecular designs. Resultingly, the microbial metabolism of these fluoroorganics usually involves unproductive or incomplete pathways that either fail to productively transform the parental compound or lead to the accumulation of potentially hazardous subproducts. Furthermore, some of these products (e.g., fluoride) may pose as biodegradation bottlenecks, by preventing the unfolding of further catabolic reactions due to their toxicity towards degrading microorganisms.

In this context, the use of microbial consortia for the biodegradation of fluorinated pesticides has the potential to yield better results than the use of single strains, as consortia can more easily showcase complementary metabolic traits and avoid the burden of centralizing complete catabolic pathways on a single microorganism [2]. A microbial consortium allows the division of labor by compartmentalizing catabolic steps on specialized strains, while also developing a syntrophic cooperation among them that maximizes the biodegradation output [3]. Additionally, the interspecies exchange of genetic resources is facilitated in a consortium, granting the whole community a higher rate of development towards desirable functional traits, including the productive catabolism of refractory pollutants such as fluorinated pesticides [4]. Nonetheless, the manipulation of microbial consortia is often a challenge due to the inherent complex microbial dynamics, individual variability of single species and the temporal instability and irreproducibility of the desired phenotype [5]. Furthermore, if the target phenotype is achieved by a wild consortium (e.g., a microbial community enriched from an environmental sample), the successful cultivation of its microbial constituents in solid media may be necessary to enable its downstream applications, which often represents a highly biasing task [3].

Epoxiconazole (EPO) and fludioxonil (FLU) (Figure S1) are two examples of fluorinated pesticides not readily degraded in the natural environment, as shown by their typically high half-lives in soils (over 353 and 218 days for EPO and FLU, respectively) [6] and by the scant literature reporting their productive biodegradation [7,8]. As a result, both these compounds have been reported to persist and accumulate in aquatic and terrestrial ecosystems [9,10,11,12]. We recently reported the successful enrichment of two bacterial consortia obtained from an agricultural soil with high capacity for the complete removal and defluorination of EPO and FLU (one consortium for each pesticide) in a wide range of pesticide concentrations, after 21 to 28 days of incubation [7]. Characterization of the cultivable microbial community of these consortia yielded a phylogenetically narrow group of bacterial strains, all accommodated within the Proteobacteria phylum, whose contribution to the observed catabolic phenotypes remained unresolved. Thus, the present work aimed to identify which of these bacterial strains are essential for the biodegradation of EPO or FLU, with the final goal of assembling an optimized version of these consortia stripped of bacterial strains that are not directly involved in the biodegradation processes. We hypothesize that the optimized consortia could constitute a robust template to design efficient bioremediation strategies for the environmental removal of these pesticides.

## 2. Materials and Methods

### 2.1. Reagents, Microorganisms, and Culture Conditions

EPO and FLU (>99% purity) were acquired from LGC Labor GmbH Augsburg Dr. Ehrenstorfer GmbH (Augsburg, Germany). Working solutions of the pesticides (5 g·L^−1^) were prepared in filter-sterilized methanol (0.2 µm cellulose acetate membrane filter) and stored at −20 °C protected from light.

Bacterial strains previously isolated from microbial consortia derived from an agricultural soil and enriched with EPO or FLU were used in this work (Table 1) [7]. Isolated strains were revived from cryogenic stasis by spreading them in the culture medium in which they were isolated, either Plate-Count agar or Minimal Salts medium agar supplemented with the respective target pesticide. Bacterial strains were routinely kept in their corresponding solid media by streaking them in fresh media every 2 weeks. Prior to each assay, bacterial strains were streaked in fresh culture medium at the same time and were allowed to grow for 48 h at 28 °C.

### 2.2. Optimization Trials Using Different Bacterial Combinations

With the objective of narrowing down the optimal bacterial composition for the biodegradation of the target fungicides, different combinations of bacterial strains isolated from the EPO or FLU-enriched consortia were tested for their ability to biodegrade their corresponding pesticides. Initially, a series of combinations were created by assembling different bacterial strains excluding only one of their corresponding isolated strains, assuming that loss of defluorination performance was indicative of the absence of a key bacterial player. Based on the results from this first trial, a second optimization round was established (only for EPO) by creating new combinations with the most promising bacterial strains. In parallel to each optimization trial, control cultures containing all the strains reassembled according to their original composition were established. For all bacterial formulations, similar cellular densities of each strain were used (optical densities at 600 nm of 0.20 ± 0.05). Each bacterial combination and respective control culture were inoculated in duplicate into 100 mL borosilicate flasks containing 30 mL of a sterile minimal salts medium (MM) [13] and supplemented with 5 mg·L^−1^ of the target fungicide (each compound dissolved in a 0.1% *v*/*v* methanolic solution). Following previously optimized conditions [7], these cultures were also fed twice a week with 400 mg·L^−1^ of sodium acetate and incubated in closed flasks for 28 days at room temperature, in static conditions and protected from light. 

### 2.3. Biodegradation by Axenic Cultures

Biodegradation by axenic cultures was only investigated for EPO. Single strains were inoculated into 100 mL borosilicate flasks containing 30 mL of MM, at an initial optical density (at 600 nm) of 0.20 ± 0.05. Each axenic culture was established in duplicate and was supplemented with 5 mg·L^−1^ of EPO (compound dissolved in a 0.1% *v*/*v* methanolic solution) and 400 mg·L^−1^ of sodium acetate (co-substrate supplemented twice a week). Cultures were incubated in closed flasks for 28 days at room temperature, in static conditions and protected from light.

### 2.4. Investigation of Growth in Sodium Acetate

The growth profile of the bacterial strains isolated from each original consortium in sodium acetate, the co-substrate used in the biodegradation experiments, was also investigated. For this, single strains were inoculated in triplicate into 100 mL borosilicate flasks, containing 30 mL of MM at an initial optical density (at 600 nm) of 0.20 ± 0.05. Cultures were supplemented once with 400 mg·L^−1^ of sodium acetate and incubated in closed flasks at room temperature, in static conditions and protected from light. Bacterial growth was analyzed by spectrophotometry at regular intervals for 48 h.

### 2.5. High-Throughput Sequencing of 16S rRNA Amplicons

The taxonomic profile of the enriched consortia from which the bacterial strains originated was analyzed, following a culture-independent approach based on the high-throughput sequencing of the V4-V5 hypervariable region of 16S rRNA amplicons. For this end, cryopreserved stocks of these consortia were cultivated in triplicate cultures on 30 mL of MM supplemented with 5 mg·L^−1^ of the target pesticide (compound dissolved in a 0.1% *v*/*v* methanolic solution) and fed twice a week with 400 mg·L^−1^ of sodium acetate. Aliquots (1 mL) of each triplicate cultures were sampled before and after a 21-day incubation period. Each sample was centrifuged (13,000× *g* for 5 min) and the resultant pellets were stored at −20 °C until DNA extraction. DNA was extracted from biomass pellets using the E.Z.N.A.^®^ Bacterial DNA Kit (Omega Bio-Tek Inc, Norcross, GA, USA) following the manufacturer’s instructions and was quantified by fluorometry using the Qubit^®^ dsDNA HS Assay Kit (Thermo Fisher Scientific, Waltham, MA, USA). DNA extracts obtained from replicates of each consortium were then pooled and their integrity was assessed by performing the amplification of the V4-V5 hypervariable region of the 16S rRNA genes by PCR, using the primers 515F-Y 5′-GTGYCAGCMGCCGCGGTAA-3′ and 926R 5′-CCGYCAATTYMTTTRAGTTT-3′ [14]. Samples showing robust amplification bands with an appropriate size (ca. 412 bp) were sent for sequencing at Genoinseq (Cantanhede, Portugal).

At Genoinseq, samples were prepared for Illumina Sequencing by 16S rRNA gene amplification of the bacterial community. The DNA was amplified for the hypervariable V4-V5 region with specific primers and further reamplified in a limited-cycle PCR reaction to add sequencing adapters and dual indexes. The first PCR reactions were performed for each sample using KAPA HiFi HotStart PCR Kit according to manufacturer’s suggestions, 0.3 μM of each PCR primer: forward primer 515F-Y and reverse primer 926R [14] and 12.5 ng of template DNA in a total volume of 25 μL. The PCR conditions involved a 3 min denaturation at 95 °C, followed by 25 cycles of 98 °C for 20 s, 50 °C for 30 s, 72 °C for 30 s, and a final extension at 72 °C for 5 min. Second PCR reactions added indexes and sequencing adapters to both ends of the amplified target region according to manufacturer’s recommendations (Illumina, San Diego, CA, USA). Negative PCR controls were included for all amplification procedures. PCR products were then one-step purified and normalized using SequalPrep 96-well plate kit (ThermoFisher Scientific, Waltham, MA, USA) [15], pooled, and pair-end sequenced in the Illumina MiSeq^®^ sequencer with the V3 chemistry, according to manufacturer’s instructions (Illumina, San Diego, CA, USA).

### 2.6. Bioinformatic Analyses

Amplicon sequencing data was extracted from Illumina MiSeq^®^ System in fastq format and quality filtered with PRINSEQ version 0.20.4 [16] to remove sequencing adapters, reads with less than 100 bases, and trim bases with an average quality lower than Q25 in a window of 5 bases. The forward and reverse reads were merged by overlapping paired-end reads with AdapterRemoval version 2.1.5 [17] using default parameters. Filtered merged amplicons in fastq format were then converted into fasta format using MOTHUR (v. 1.43.0) [18].

High-quality sequences were then taxonomically classified through the NGS analysis pipeline of the SILVA rRNA gene database project (SILVAngs 1.3) [19]. Each read was aligned using the SILVA Incremental Aligner (SINA SINA v1.2.10 for ARB SVN (revision 21008)) [20] against the SILVA SSU rRNA SEED and quality controlled [19]. Reads shorter than 50 aligned nucleotides and reads with more than 2% of ambiguities or 2% of homopolymers, respectively, were excluded from further processing. Putative contaminations and artefacts, reads with a low alignment quality (50 alignment identity, 40 alignment score reported by SINA), were identified and excluded from downstream analysis. After these initial steps of quality control, identical reads were identified (dereplication), the unique reads were clustered into Operational Taxonomic Units (OTUs), on a per sample basis, and the reference read of each OTU was classified. Dereplication and clustering was done using cd-hit-est (version 3.1.2; http://www.bioinformatics.org/cd-hit; accessed on 5 October 2019) [21] running in accurate mode, ignoring overhangs, and applying identity criteria of 1.00 and 0.98, respectively. The classification was performed by a local nucleotide BLAST search against the non-redundant version of the SILVA SSU Ref dataset (release 132; http://www.arb-silva.de; accessed on 5 October 2019) using blastn (version 2.2.30+; http://blast.ncbi.nlm.nih.gov/Blast.cgi; accessed on 5 October 2019) with standard settings [22]. The classification of each OTU reference read was mapped onto all reads that were assigned to the respective OTU. This yields quantitative information (number of individual reads per taxonomic path), within the limitations of PCR and sequencing technique biases, as well as multiple rRNA operons. Reads without any BLAST hits or reads with weak BLAST hits, where the function “(% sequence identity + % alignment coverage)/2” did not exceed the value of 93, remained unclassified, and was assigned to the meta group “No Relative” in the SILVAngs fingerprint.

In parallel, local BLAST analyses were performed to retrieve the 16S rRNA gene sequences of the bacterial strains listed in Table 1 from the generated 16S rRNA amplicon datasets. For this, the V4-V5 regions were extracted from the full-length 16S rRNA genes of all bacterial isolates, aligned using the Geneious software (v11.1.4), and then were used as reference sequences of a custom dataset. Separately, the 16S rRNA amplicons obtained from the Illumina MiSeq platform were processed using MOTHUR (v.1.43.0) [18]. Both the raw forward and reverse fastq files were first merged, then filtered by removing all merged reads containing non-templated bases (N) and/or homopolymers. Those shorter than 300 bp were excluded. Remaining reads were dereplicated (based on 100% similarity) and, after this step, chimeras were identified de novo and removed with UCHIME [23]. The obtained unique reads were then queried against the custom database using standalone BLAST in BLAST+ suite [22,24] and only the hits with a similarity ≥99% and with a query coverage against the references sequences >300 bp were considered for the analysis.

### 2.7. Analytical Methods

Cellular optical densities were determined by spectrophotometry, by reading the absorbance of culture aliquots at 600 nm in a V-1200 spectrophotometer (VWR International, LLC, Philadelphia, PA, USA). Defluorination of the target pesticides was used as a biodegradation indicator and was determined through potentiometric quantification of the fluoride anion in the cultures supernatants, as described in Alexandrino et al. [13].

## 3. Results

### 3.1. Biodegradation of EPO and FLU Using Different Bacterial Combinations

In an attempt to optimize the bacterial composition of two consortia capable of degrading EPO and FLU, bacterial strains previously isolated from microbial cultures obtained from an agricultural soil through enrichment with EPO or FLU [7] were assembled in different combinations and their defluorination efficiencies were monitored over time.

**Figure 1:**
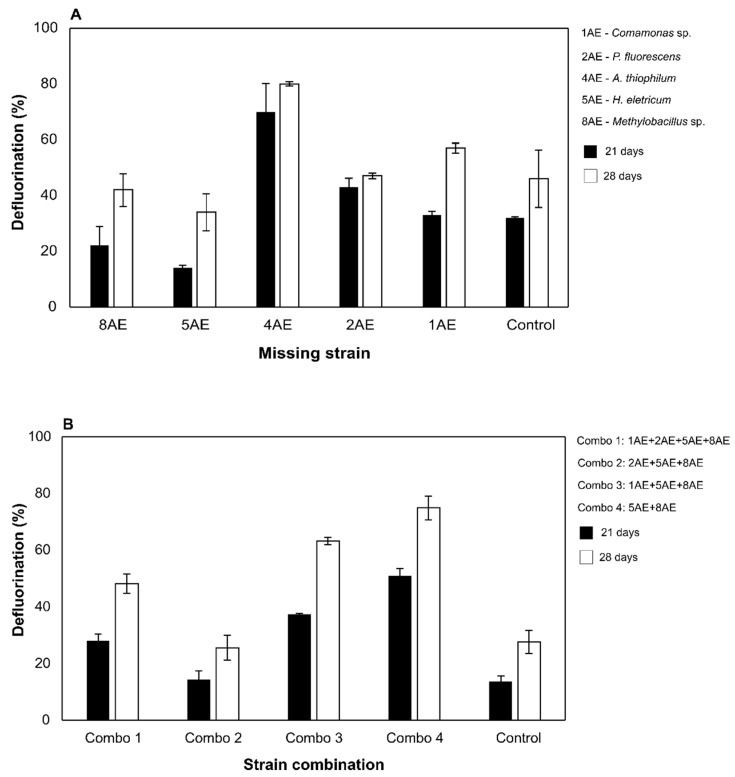
Results from the first (panel **A**) and second (panel **B**) optimization trials conducted with the bacterial strains isolated from the EPO-enriched consortium, after 21 and 28 days of incubation. Control cultures corresponded to a mixture of all bacterial isolates tested in both assays. Results are expressed as the mean of duplicates and bars represent the error.

For EPO, a total of five taxonomically distinct bacterial strains were used in the assays (Table 1). In the first optimization trial, a series of combinations were made by assembling different strains excluding only one of their corresponding isolated strains. Defluorination of EPO was detected in all the tested combinations, though with varying efficiencies (Figure 1A). The bacterial combination missing the strain *A. thiophilum* 4AE stood out as the best performing one, being capable of achieving about 80% of EPO defluorination after 28 days (Figure 1A). On the other hand, the combinations missing *H. eletricum* 5AE or *Methylobacillus* sp. 8AE presented the lowest defluorination performances (Figure 1A). Based on these results, a second optimization trial combining the most promising strains from the first optimization trial was carried out. Results showed that the combination composed by *H. eletricum* 5AE and *Methylobacillus* sp. 8AE (Combo 4) had the best performance, defluorinating ca. 80% of EPO after 28 days (Figure 1B). Surprisingly, in this second optimization trial, both the control cultures and the bacterial combination missing *A. thiophilum* 4AE (Combo 1) showed lower defluorinations than in the first assay (Figure 1).

**Figure 2:**
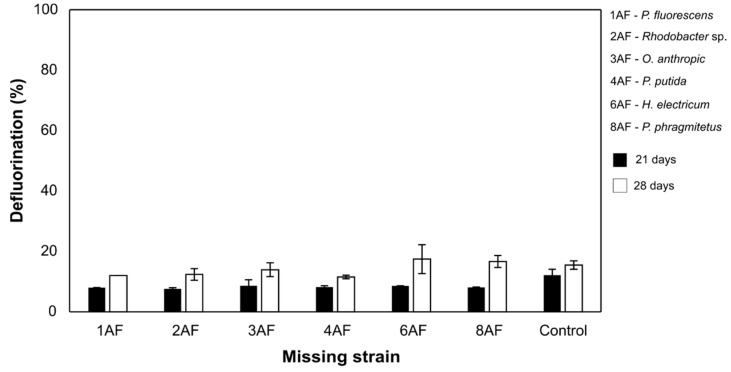
Results from the optimization trial conducted with the bacterial strains isolated from the FLU-enriched consortium, after 21 and 28 days of incubation. Control cultures corresponded to a mixture of all bacterial isolates tested in the assay. Results are expressed as the mean of duplicates and bars represent the error.

Concerning FLU, six taxonomically different bacterial strains were used for the optimization trials (Table 1), which also started by testing different bacterial formulations missing only one strain. However, the biodegradation performances obtained for this pesticide were drastically lower than those of their consortium of origin, which was capable of completely defluorinating FLU during the same incubation period [7], with none of the tested combinations achieving defluorination efficiencies higher than 20% (Figure 2).

### 3.2. Biodegradation of EPO by Axenic Cultures

**Figure 3:**
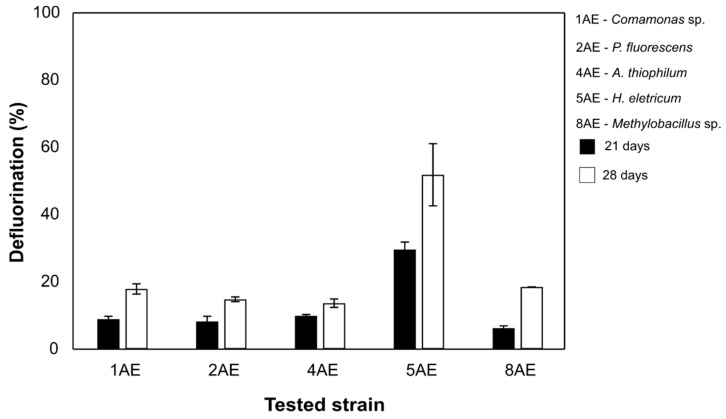
Defluorination percentage of each bacterial strain isolated from the EPO-enriched consortium, after 21 and 28 days. Results are expressed as the mean of duplicates and bars represent the error.

Given the poor defluorination performances exhibited by the different strains combinations tested for FLU, we decided to investigate biodegradation by axenic cultures only for EPO. In this assay, none of the tested bacterial strains were able to replicate the catabolic performances observed in the optimization trials with the different bacterial combinations. Still, *H. eletricum* 5AE displayed a promising catabolic performance by defluorinating 52 ± 9% of the fungicide after 28 days of incubation (Figure 3). The remaining bacterial strains showed EPO defluorination efficiencies that never exceeded 20% during the same incubation period (Figure 3).

### 3.3. Growth of Bacterial Strains in Sodium Acetate

To understand how supplementation of sodium acetate (used as co-substrate in the biodegradation experiments of the pesticides) can influence the growth of the strains retrieved from the original consortia enriched with EPO and FLU, the growth pattern of these strains when only fed with this co-substrate was investigated. This influence was experimentally determined in the same oligotrophic saline medium used in the optimization trials, where heterotrophic growth can only be achieved through the exogenous supplementation of suitable growth substrates (e.g., sodium acetate, methanol, or the target fungicides).

The tested strains exhibited different growth patterns (Figure S2), allowing to discriminate a group of faster-growing strains (reaching peak growth after 24 h of co-substrate supplementation) and slower-growing strains (peaking after 32 to 48 h of co-substrate supplementation). The group of faster-growing strains was composed by *A. thiophillum* 4AE, *H. eletricum* 5AE and *P. phragmitetus* 8AF, which achieved different peak cellular densities (Figure S2). The remaining strains achieved their peak growth between 32 and 48 h, with some of them showing only a slight growth in sodium acetate during the incubation period, such as *P. fluorescens* 2AE, *P. fluorescens* 1AF and *Rhodobacter* sp. 2AF (Figure S2). *Methylobacillus sp*. 8AE was the only tested strain unable to use sodium acetate as a growth substrate (Figure S2).

### 3.4. Bacterial Structure of the Original Enriched Consortia

With the objective of unravelling the bacterial structure of the consortia enriched with EPO and FLU from which the bacterial strains used in this study were isolated, a metabarcoding approach targeting a 16S rRNA biomarker was followed. This analysis was carried out at the beginning and the end of a 21-day incubation cycle, during which the original consortia enriched with EPO and FLU were able to completely defluorinate 5 mg·L^−1^ of their corresponding fungicide. This approach allowed us to understand how the retrieved isolated strains were represented in the whole community, while also detecting relevant bacterial shifts resultant from the biodegradation process and inferring if the key degrading bacteria were successfully isolated.

**Figure 4:**
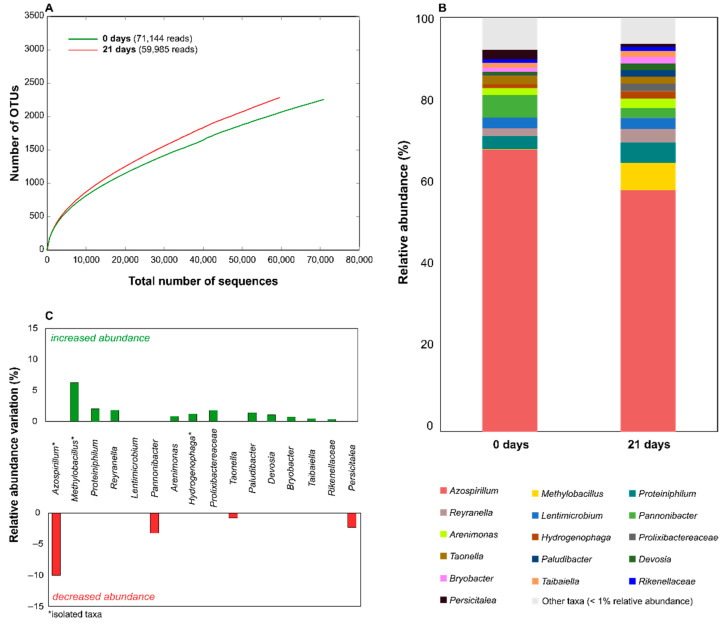
16S rRNA amplicon sequencing data for the EPO-enriched consortium at the beginning and at the end of a 21-day incubation period with 5 mg·L^−1^ of EPO. Figure panels show the alpha rarefaction curves (panel **A**), the bacterial consortium structure at the family or genus levels (panel **B**), and the relative abundance variations observed during the 21-day incubation period with EPO (panel **C**). The number of amplicon reads obtained for each bacterial taxa is shown in Table S1.

**Figure 5:**
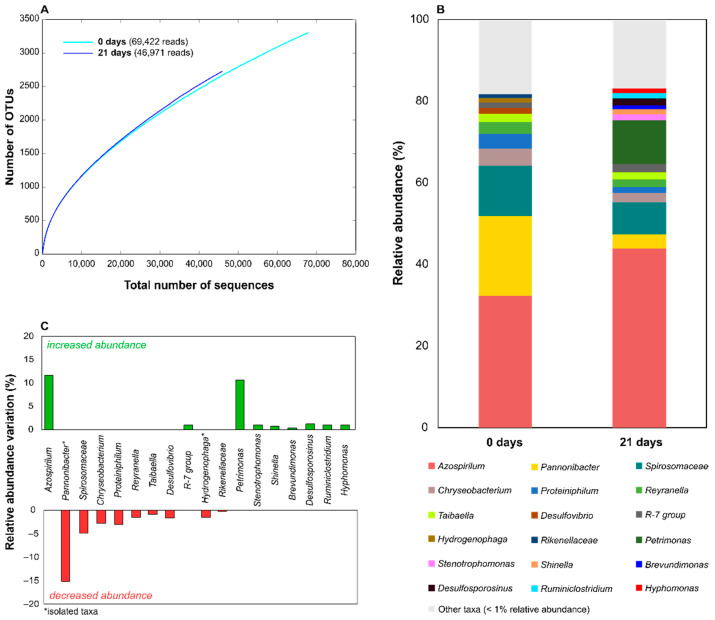
16S rRNA amplicon sequencing data for the FLU-enriched consortium at the beginning and at the end of a 21-day incubation period with 5 mg·L^−1^ of FLU. Figure panels show the alpha rarefaction curves (panel **A**), the bacterial consortium structure at the family or genus levels (panel **B**), and the relative abundance variations observed during the 21-day incubation period with FLU (panel **C**). The number of amplicon reads obtained for each bacterial taxa is shown in Table S2.

The sequencing effort produced a total of 247,522 sequences, 241,096 of which (97.40%) were taxonomically classified. For the EPO-enriched consortium, the number of classified reads per sample ranged between 59,985 (0 days) and 71,144 (21 days), while for the FLU-enriched consortium they varied between 46,971 (0 days) and 69,422 (21 days) (Figure 4 and Figure 5). The taxonomic profile of the EPO-enriched consortium revealed that the strains retrieved from this consortium and used in this study represented a small subset of the whole bacterial diversity of the consortium (Table 2). Although this community was dominated by *Azospirillum* taxa during the biodegradation process (Figure 4), the isolate *A. thiophillum* 4AE represented a small fraction of this genus (Table 2). Contrarily, the isolates *H. eletricum* 5AE and *Methylobacillus* sp. 8AE were the sole representatives of their genera in the consortium (Table 2). The representation of these latter taxa in the consortium increased after the 21-day incubation period with EPO, a trend that was also shared by several other phylotypes that were not isolated, such as *Proteiniphillum, Reyranella*, or *Prolixibactereaceae* (Figure 4). Interestingly, although *Azospirillum* remained the predominant genus throughout the biodegradation process, its representation in the consortium decreased after the 21-day incubation period, which was also observed for the taxa *Pannonibacter*, *Taonella*, and *Persicitaleae* (Figure 4).

For the EPO-enriched consortium, the bacterial strains isolated from the FLU-enriched consortium represented a small fraction of its diversity (Table 2). However, this community revealed a distinct taxonomic structure other than the EPO-enriched consortium, as it was not dominated by a single phylotype, but instead was co-dominated by *Azospirillum*, *Pannonibacter*, and *Spirosomaceae* bacteria (Figure 5). The dynamics of this co-dominance shifted during the biodegradation process, with *Azospirillum* and *Petrimonas* exhibiting a higher representation after the 21-day incubation period, with the latter genus becoming the third most represented taxa in the consortium alongside *Azospirillum* and *Spirosomaceae* (Figure 5). On the other hand, *Pannonibacter* and *Spirosomaceae* showed a decreasing trend during this period (Figure 5). Regardless, all the isolated strains were found to have a slim representation in the consortium (Table 2), despite some of the bacterial taxa where they are accommodated having a relevant representation in the consortium (e.g., *Pannonibacter*) (Figure 5).

## 4. Discussion

The microbial metabolism of EPO and FLU has remained elusive for several years, with the few studies on this topic suggesting that environmental microorganisms do not play a direct role in the environmental depuration of these pesticides [25,26]. Indeed, these compounds exhibit outstanding resistance to both biotic and abiotic degradation mechanisms, having shown to be prone to persist in soils, sediments, and biomixtures [27,28,29,30,31]. Nonetheless, we recently reported the biodegradation of these pesticides by environmental microbial communities obtained through a selective enrichment period [7]. We were also able to isolate several bacterial strains from these enriched consortia, but the contribution of each strain to the biodegradation of EPO and FLU was unclear, which triggered the work presented in this study. Here, culture-dependent and independent approaches were combined as an attempt to achieve an optimized version of these degrading consortia and obtain insights on the strains that could be more relevant for the biodegradation process. As such, different combinations of the bacterial strains isolated from the EPO and FLU-enriched consortia were created and their capacity to biodegrade these pesticides was investigated. Defluorination was used as a key biodegradation indicator, as we previously showed that this catabolic step is exclusively mediated by microorganisms under the incubation conditions adopted in the present study [7].

The bacterial strains isolated from the FLU-enriched consortium displayed limited efficiencies in the defluorination of this fungicide, even when all the isolated strains were combined. This drastically contrasted with the catabolic performance of their consortium of origin, which was able to defluorinate up to 10 mg·L^−1^ of FLU in 12 days [7], suggesting that the key bacterial degraders of the consortium were not successfully isolated. Although it is possible that these isolated strains can metabolize FLU via pathways not involving defluorination reactions, these would still lead to the production and accumulation of fluorinated subproducts potentially hazardous to the environment [32], and this is not the feature that we were looking for.

On the other hand, the results obtained in the first optimization trial using the strains isolated from the EPO-enriched consortium suggested that *H. eletricum* 5AE and *Methylobacillus* sp. 8AE could have a relevant role in the biodegradation of EPO, given that the omission of these strains from the bacterial combinations led to the lowest defluorination performances. This hypothesis was corroborated when a co-culture of both these bacterial species yielded a defluorination output closely resembling their consortium of origin. The investigation of the defluorination performances of these strains as axenic cultures revealed that *H. eletricum* 5AE was able to defluorinate EPO as a single strain, though less efficiently than when in combination with *Methylobacillus* sp. 8AE, while this latter strain defluorinated only a small percentage of EPO under the same conditions. This reveals an interesting dynamic between both strains, in which *H. eletricum* 5AE seems to have a more prevalent role in the defluorination of EPO and *Methylobacillus* sp. 8AE seems to play a complementary role in the degradation process. These results also show that both strains can withstand the accumulation of fluoride resulting from the productive defluorination of EPO, which is well within the concentration thresholds usually linked to fluoride toxicity in bacterial cells [33]. Given its methylotrophic nature, *Methylobacillus* sp. 8AE can rapidly consume the methanol used as the EPO solvent, achieving the stationary growth phase after 72 h when supplemented with a 0.1% *v*/*v* of methanolic solution (data not shown). However, the taxonomic profile of the original consortium enriched with EPO revealed that the genus *Methylobacillus*, which was solely represented by strain 8AE, increased its abundance in the original consortium during the biodegradation process, which could only be achieved if suitable one-carbon growth substrates were consistently available to the strain during the 21-days incubation period. Given the inability of *Methylobacillus* sp. 8AE to grow on the supplemented co-substrate, sodium acetate, this suggests that this strain incorporated carbon not only from the supplied methanol but might also be growing at the expense of one-carbon moieties from EPO or sub-products resultant from the biodegradation of this pesticide. Indeed, carbon assimilation could have occurred directly, possibly via the oxidative demethylation of EPO moieties lacking C-C bonds [34], such as its epoxy or triazole functional groups, or indirectly, through a syntrophic relationship with *H. eletricum* 5AE, in which strain 8AE could have consumed one-carbon by-products derived from the defluorination of EPO by strain 5AE. Nonetheless, the fact that the exclusion of *Methylobacillus* sp. 8AE from the bacterial combinations resulted in a decrease in EPO defluorination suggests a more active role of this bacterium in the catabolic process, perhaps indicating that this strain catalyzes the formation of subproducts more susceptible for defluorination, thus favoring the former hypothesis. In fact, the capacity of several methylotrophic bacteria, including some *Methylobacillus* species, to take advantage of their methylotrophy to attack complex xenobiotic substrates has been shown before [35,36].

Results from the optimization trials also showed that the exclusion of *A. thiophilum* 4AE from the bacterial combinations led to improved defluorination efficiencies, suggesting a possible inhibitory effect of this strain on the defluorination of EPO. Given that the original consortium can efficiently metabolize EPO, despite the dominance of *Azospirillum* bacteria, suggests that this catabolic impairment is resultant from the specific presence of *A. thiophilum* 4AE in the culture. *A. thiophilum* 4AE had a high growth performance on sodium acetate, far superior to that shown for its bacterial cohorts, which may allow this strain to become predominant in the consortium when present as the sole *Azospirillum* representative. This predominance may not occur when this strain co-exists with other *Azospirillum* bacteria [37], which would explain why this inhibitory effect is not observed in the original consortium where this particular strain has poor representation. Such voracity for sodium acetate consumption may have allowed *A. thiophilum* 4AE to rapidly achieve cellular densities high enough to disrupt the defluorination of EPO, possibly due to substrate competition or other opportunistic behaviors, not allowing the proliferation of the strains more relevant for EPO biodegradation. Still, regardless of these dynamics, it was clear that the defluorination of EPO, a key step in the productive biotransformation of fluorinated pesticides, can be achieved by minor phylogenetic groups present in the consortium with a cumulative relative abundance of ca. 10%. While it is possible that other bacterial players that could not be isolated from the original enriched consortium can also contribute to the biodegradation of EPO, the findings of this work emphasize the importance that low abundant taxa may have in the productivity of microbial communities, particularly when biodegradation of persistent pollutants is concerned. The contribution of low abundant or even rare bacterial taxa for the biodegradation of xenobiotics is often disregarded [38], but this work contributes to highlight the role of minor bacterial groups in the dehalogenation of relevant, often highly recalcitrant, haloorganics [39,40].

This work also showed that EPO and FLU caused distinct selective pressures on the environmental microbial communities. Despite exhibiting similar taxonomic compositions, which was expected due to their common environmental origin (agricultural soil) [7], the presence of EPO or FLU led to distinct community structures, which may explain why very few bacterial isolates shared a phylogenetic relationship between the two consortia. The exceptions to this were *P. fluorescens* and *H. eletricum*, which were both recovered from the consortia enriched with EPO and FLU. A comparison of the complete 16S rRNA sequences of the strains sharing the same taxonomy revealed full sequence homology among them (data not shown), indicating that strains 1AF and 2AE, as well as strains 6AF and 5AE, correspond, respectively, to the same *P. fluorescens and H. eletricum* species. In the case of the latter species, this result suggests that *H*. *eletricum* may have differing catabolic roles during the biodegradation of EPO and FLU. Furthermore, the elucidation of the taxonomic structure of the EPO and FLU-enriched consortia was paramount to understand the slim taxonomic representation of the isolated bacterial strains in their consortium of origin and, consequently, the limitations of cultivation approaches. While this cultivation bias did not affect so much the recovery of strains with a role on the biodegradation of EPO, it led to the unsuccessful recovery of bacterial strains capable of efficiently defluorinating FLU. Limitations on the recovery of bacterial species by conventional culture-dependent methodologies are naturally expected, given the impossibility of catering to the ideal growth conditions of all bacteria present in a complex community. This difficulty increases several-fold when the reconstruction of a wild consortium exhibiting atypical catabolic phenotypes, often reliant on sensitive and/or transient microbial dynamics, is required, as shown before [41]. Nevertheless, these results emphasize the importance of adequate cultivation approaches in the engineering of microbial consortia for bioremediation purposes and the need to improve current culture-dependent strategies to maximize the recovery of catabolically relevant microorganisms.

## 5. Conclusions

This work contributed to clarify the role of the bacterial strains previously isolated from two consortia enriched with EPO and FLU in the biodegradation of these pesticides. Our results suggest that strains *H. eletricum* 5AE and *Methylobacillus* sp. 8AE act cooperatively for the defluorination of EPO, given the high defluorination performance obtained when only these strains were present in co-culture. Though the contribution of non-isolated bacterial species should not be ruled out, these results indicate that biodegradation of this persistent pesticide can be led by minor phylotypes, given the slim representation of *H. eletricum* 5AE and *Methylobacillus* sp. 8AE in the original enriched consortium, highlighting the role of low abundant bacterial taxa in the biodegradation of persistent pesticides. In such a way, this work reveals, for the first time, the direct involvement of specific bacterial isolates in the biodegradation of EPO. As for FLU, the results obtained revealed that none of the tested bacterial strains could catalyze the efficient defluorination of this pesticide, which emphasizes the importance of adequate culture-dependent approaches in the engineering of microbial consortia for pesticide bioremediation.

## Figures and Tables

**Table 1:** Previously isolated bacterial strains used in the optimization trials [7]. EPO—Epoxiconazole; FLU—Fludioxonil; PCA—Plate-Count agar; MM—Minimal Salts Medium.

Strain	Taxonomy	Target Fungicide	Culture Medium	GenBank^®^ Accession Number
1AE	*Comamonas* sp.	EPO	PCA	MK127552
2AE	*Pseudomonas fluorescens*	EPO	PCA	MK127553
4AE	*Azospirillum thiophilum*	EPO	PCA	MK128510
5AE	*Hydrogenophaga eletricum*	EPO	PCA	MK128663
8AE	*Methylobacillus* sp.	EPO	MM with EPO	MK128665
1AF	*Pseudomonas fluorescens*	FLU	PCA	MK128959
2AF	*Rhodobacter* sp.	FLU	PCA	MK128963
3AF	*Ochrobactrum anthropic*	FLU	PCA	MK128962
4AF	*Pseudomonas putida*	FLU	PCA	MK128965
6AF	*Hydrogenophaga electricum*	FLU	MM with FLU	MK128969
8AF	*Pannonibacter phragmitetus*	FLU	MM with FLU	MN853325

**Table 2:** Representation percentage (%) of the bacterial strains isolated from the consortia enriched with EPO (AE) and FLU (AF) in these consortia and in the corresponding genus, at the end of a 21-day incubation period, based on local BLAST analyses.

Isolated Strain	Representation in the Genus	Representation in the Consortium
*Comamonas* sp. 1AE	100	<1
*Pseudomonas fluorescens* 2AE	n/a *	n/a *
*Azospirillum thiophilum* 4AE	1.8	1.1
*Hydrogenophaga eletricum* 5AE	100	2.8
*Methylobacillus* sp. 8AE	100	6.6
*Pseudomonas fluorescens* 1AF	n/a *	n/a *
*Rhodobacter* sp. 2AF	100	<1
*Ochrobactrum anthropic* 3AF	13.5	<1
*Pseudomonas putida* 4AF	30.8	<1
*Hydrogenophaga electricum* 6AF	100	<1
*Pannonibacter phragmitetus* 8AF	<1	<1

* The high homology observed between the 16S rRNA V4-V5 sequences of *P. fluorescens* 1AF and 2AE and the other *Pseudomonas* in the consortia precluded the accurate quantification of amplicon reads specific to these strains.

## Data Availability

Not applicable.

## Supplementary Materials

The following are available online at www.mdpi.com/article/10.3390/microorganisms9102109/s1, Figure S1: Chemical structure of the target fungicides; Figure S2: Growth of the bacterial isolates retrieved from the EPO and FLU-enriched consortia on 400 mg·L^−1^ of sodium acetate. Results are expressed as the mean of triplicates and error bars represent standard deviation. Lines connecting data points are just for guiding purposes.; Table S1: Number of reads obtained for each OTU representing ≥ 1% of the relative abundance of the EPO-enriched consortium, either at the beginning (0 days) or at the end of the incubation period (21 days); Table S2: Number of reads obtained for each OTU representing ≥ 1% of the relative abundance of the FLU-enriched consortium, either at the beginning (0 days) or at the end of the incubation period (21 days).
