# Supplementary material for: Combining Culture-Dependent and Independent Approaches for the Optimization of Epoxiconazole and Fludioxonil-Degrading Bacterial Consortia
Source: Microorganisms. 2021 Oct 7;9(10):2109. doi: 10.3390/microorganisms9102109 (PMC8538489; doi:10.3390/microorganisms9102109)
Supplement: Supplementary file 1 [file microorganisms-09-02109-s001.zip › microorganisms-1380303-supplementary.pdf]

Figure S1. Chemical structure of the target fungicides.

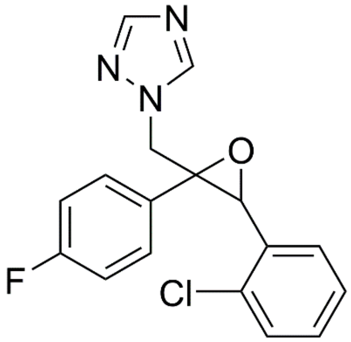

Epoxiconazole

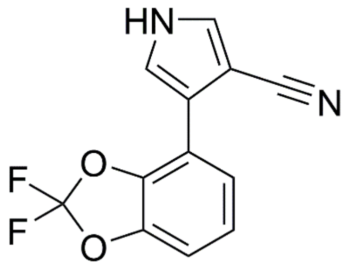

Fludioxonil

Figure S2. Growth of the bacterial isolates retrieved from the EPO and FLU-enriched consortia on 400 mgL^{-1} of sodium acetate. Results are expressed as the mean of triplicates and error bars represent standard deviation. Lines connecting data points are just for guiding purposes.

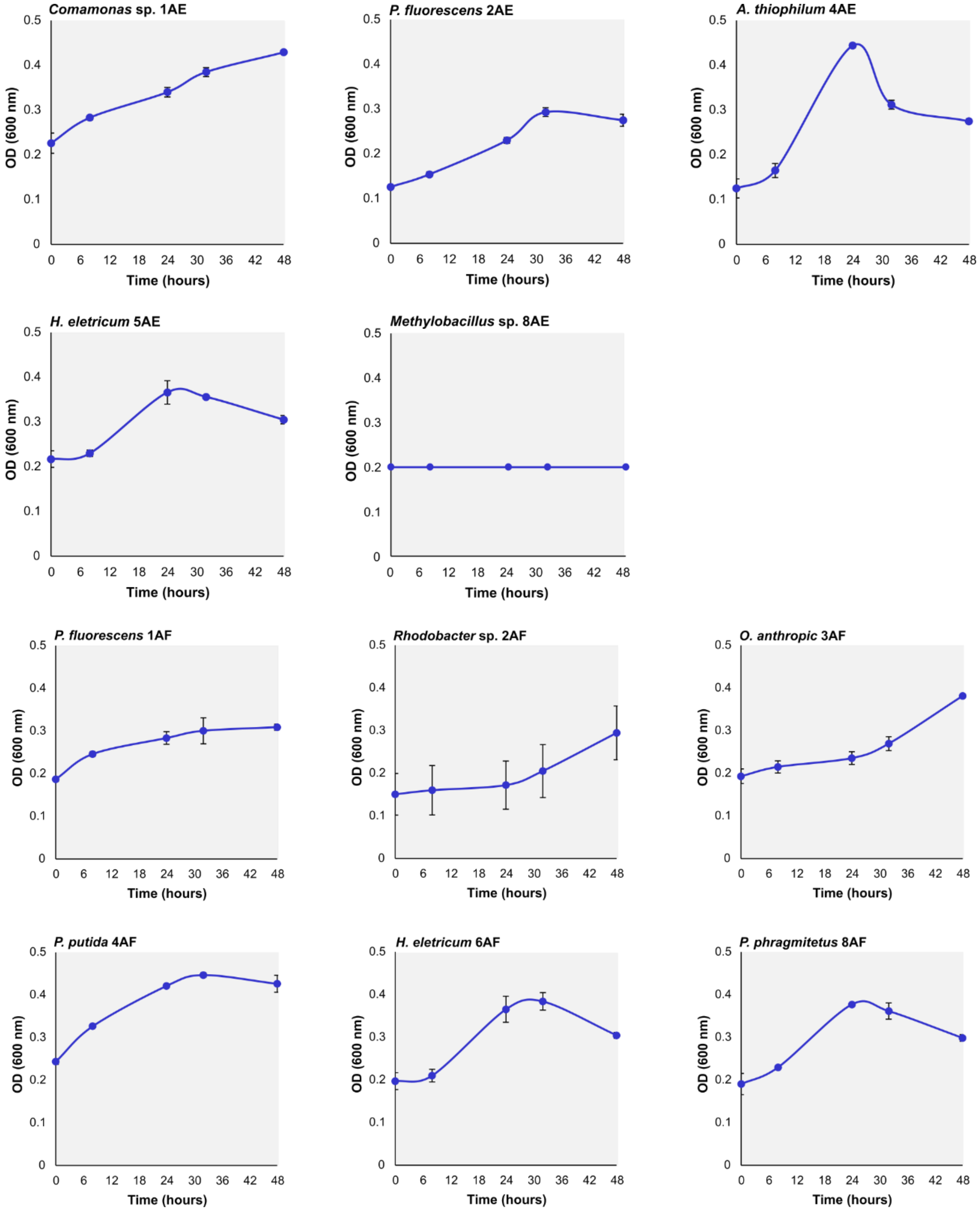

Table S1. Number of reads obtained for each OTU representing ≥ 1% of the relative abundance of the EPO-enriched consortium, either at the beginning (0 days) or at the end of the incubation period (21 days)

**OTU taxonomy**	**Number of reads**	
	0 days	21 days
Azospirillum	48358	34953
Methylobacillus	122	3983
Proteiniphilum	2297	2912
Reyranella	1261	2003
Lentimicrobium	1830	1555
Pannonibacter	3961	1452
Arenimonas	1128	1324
Hydrogenophaga	656	1699
Prolixibactereaceae	54	1049
Taonella	1451	1025
Paludibacter	185	1014
Devosia	480	930
Bryobacter	701	915
Taibaiella	844	884
Rikenellaceae	558	614
Persicitalea	1672	414

Table S2. Number of reads obtained for each OTU representing ≥ 1% of the relative abundance of the FLU-enriched consortium, either at the beginning (0 days) or at the end of the incubation period (21 days)

**OTU taxonomy**	**Number of reads**	
	0 days	21 days
Azospirilum	22390	20611
Pannonibacter	13626	1627
Spirosomaceae	8524	3692
Chryseobacterium	2921	1056
Proteiniphilum	2531	683
Reyranella	1948	898
Taibaella	1406	825
Desulfovibrio	1005	683
R-7 group	961	942
Hydrogenophaga	745	3
Rikenellaceae	731	132
Petrimonas	559	5041
Stenotrophomonas	417	681
Shinella	132	570
Brevundimonas	392	475
Desulfosporosinus	782	1005
Ruminiclostridium	584	744
Hyphomonas	160	544
